# Metabolomic Changes in *Sogatella furcifera* under *Southern rice black-streaked dwarf virus* Infection and Temperature Stress

**DOI:** 10.3390/v10070344

**Published:** 2018-06-26

**Authors:** Tong Zhang, Wendi Feng, Jiajie Ye, Zhanbiao Li, Guohui Zhou

**Affiliations:** Guangdong Province Key Laboratory of Microbial Signals and Disease Control, College of Agriculture, South China Agricultural University, Guangzhou 510642, China; zhangtong@scau.edu.cn (T.Z.); fengwendis@163.com (W.F.); yejiajie_alin@163.com (J.Y.); lizhanbizo8410@sina.com (Z.L.)

**Keywords:** white-backed planthopper (WBPH), *Southern rice black-streaked dwarf virus* (SRBSDV), temperature stress, gas chromatography-time of flight-mass spectrometry (GC-TOF-MS), differential metabolite accumulation

## Abstract

*Southern rice black-streaked dwarf virus* (SRBSDV) is a devastating newly emerged rice reovirus in Eastern and Southeastern Asia transmitted by a long-distance migratory pest, the white-backed planthopper (WBPH). We previously showed that SRBSDV infection decreased the cold tolerance but improved the heat tolerance of its vector, WBPH. Comparative metabolomic analysis was used to explore the potential mechanisms underlying these changes in temperature stress response. Fourth-generation WBPH nymphs were treated with SRBSDV and/or extreme temperature stress and were analyzed using gas chromatography-time of flight-mass spectrometry. A total of 605 distinguishable peaks were identified and 165, 207, and 202 differentially accumulated metabolites were identified in WBPH after virus infection, cold, or heat stress, respectively. The nucleic acids and fatty acids were the major categories of metabolites regulated by SRBSDV infection, whereas temperature stress regulated tricarboxylic acid cycle compounds, sugars, and polyols. For the WBPH samples infected with SRBSDV and subjected to temperature stress, amino acids, sugars, and polyols were the most significant regulated metabolites. The metabolomics study suggests that SRBSDV may influence the extreme temperature tolerance of WBPH by regulating the accumulation of amino acids, sugars, and polyols in the insect body.

## 1. Introduction

*Southern rice black-streaked dwarf virus* (SRBSDV) is a newly emerged rice fjivirus species (family *Reoviridae*) that is efficiently transmitted by the white-backed planthopper (WBPH, *Sogatella furcifera*), a long-distance migratory pest, in a persistent, circulative, and propagative manner [1,2]. Since its outbreak in 2009, SRBSDV has spread over vast areas, posing a significant threat to rice production in Eastern and Southeastern Asia [1,2]. Occurrence and distribution of this viral disease have been highly correlated with the spread and migration of viruliferous WBPH [2]. Annually, populations of WBPH overwinter in Vietnam and Southern China in the Hainan and Yunnan provinces. In spring, these populations migrate northward over 1000 km via the southwest monsoon to Northern China, Japan, and Korea, before propagating and returning to their overwintering regions in late August when the monsoon direction reverses. The susceptibility to both cold and hot climates results in the long-distance migratory behavior of WBPH [2,3].

Insect vectors may benefit from the endosymbionts that provide necessary nutrient substances and confer tolerance to stresses or the harmful effects caused by pathogen infection [4,5,6]. Many insects are vectors of a variety of plant viruses, and studies have shown that plant viruses can both cause pathological changes in their hosts and regulate the adaptability of their insect vectors [7,8,9,10,11]. In terms of the interactions between the plant, virus, and vector, viruses can influence the temperature tolerance of their vectors [11,12]. This may affect the geographic distribution and perniciousness of these pests and the subsequent spread of the plant virus. Therefore, investigating the mechanism underlying the temperature adaptation of insects mediated by their vectored viruses is of considerable ecological significance.

Previous studies showed that SRBSDV infection of *S. furcifera* may strongly influence the insect’s physiology and behavior, including a prolonged nymph period, decreased fecundity, shortened adult lifespan, and altered host preference [2,13,14,15,16]. In our previous study, SRBSDV-infected or virus-free fourth and fifth instar WBPH nymphs were placed under 5 and 36 °C conditions for 48 h, with a parallel test at a suitable temperature of 25 °C as the control. At 12, 24, 36, and 48 h after extreme temperature treatment, compared to the virus-free insects, the SRBSDV-infected WBPH had significantly higher death rates after cold treatment, but significantly lower death rates under heat stress. These results indicate that SRBSDV infection in *S. furcifera* can increase the vector’s death rate under extreme cold stress but improve its survival rate under extreme heat stress [17]. Through comparative transcriptome analysis, different gene regulation patterns in WBPH under viral and/or temperature stresses were examined, and many genes involved in metabolism were found to be regulated by the stresses [17]. Thus, in the current study, we performed metabolomics analyses to determine the mechanism underlying the effects of viral infection on the temperature adaptation of its insect vector.

## 2. Materials and Methods

### 2.1. Rice Plant Culturing and Maintenance of SRBSDV

The rice plants (cultivar TaiChung Native-1, TN1) used in this study were grown as previously described [16]. The SRBSDV isolates were maintained on several rice plants grown in an insect-proof greenhouse in our laboratory. The SRBSDV-infected rice plants were identified by real-time polymerase chain reaction (RT-PCR) as previously described [16,17].

### 2.2. S. furcifera Propagation

To generate a population with minimum genetic diversity, the WBPHs used in this study were propagated to the fourth generation as previously described [17]. Some of the newly hatched, fourth-generation nymphs were transferred to SRBSDV-infected rice plants to feed for 2 days before being moved back to healthy plants and reared to fourth- to fifth-instar nymphs. The virus-fed insects and the virus-free control insects that had not fed on infected plants were then used for temperature stress treatment. The virus-carrying rates for each treatment group were confirmed using RT-PCR detection as previously described (Table S1) [17].

### 2.3. Temperature Stress Treatment for S. furcifera

The WBPH nymphs were reared in nylon gauze-sealed glass tubes (10 individuals per tube) that were incubated at different temperatures in climate chamber. The cold stress and heat stress tests were conducted at 5 and 36 °C, respectively, with a parallel test at 25 °C used as the control. After 4 h incubation, the nymphs of each replicate were pooled together and rapidly frozen in liquid nitrogen. Each test contained six replicates and each replicate contained 100 virus-fed or 100 virus-free fourth- to fifth-instar WBPH nymphs.

### 2.4. Extraction of WBPH Metabolites

All samples were mechanically ground under freezing conditions and then 60 mg of each sample was used for metabolite extraction. Briefly, each sample was extracted with 0.48 mL extraction buffer (V_methanol_:V_chloroform_ = 3:1) with 24 μL of l-2-Chlorophenylalanine (1 mg/mL stock in sterile H_2_O) added as an internal standard, vortexed for 30 s, and ultrasound treated for 6 min while incubated in ice water. After centrifugation at 12,000× *g* rpm for 15 min, the supernatants were transferred to glass vials and dried in a vacuum concentrator without heating. Next, 80 μL methoxyamination reagent (20 mg/mL in pyridine) was added and incubated for 20 min at 80 °C before 100 μL BSTFA regent (1% TMCS, *v*/*v*) was quickly added and incubated for 1 h at 70 °C. Finally, the samples were mixed well and were analyzed using gas chromatography-mass spectrometry (GC-MS).

### 2.5. GC-TOF-MS

Gas chromatography-time of flight-mass spectrometry (GC-TOF-MS) analysis was performed using an Agilent 7890 gas chromatograph system coupled with a LECO Pegasus HT time-of-flight mass spectrometer. Compounds were separated and characterized on a DB-5MS capillary column (30 m × 250 μm inner diameter, 0.25 μm film thickness; J&W Scientific, Folsom, CA, USA). A 1-μL aliquot of the analyte was injected in splitless mode. Helium was used as the carrier gas, the front inlet purge flow was 3 mL·min^−1^, and the gas flow rate through the column was 1 mL·min^−1^. The initial temperature was maintained at 50 °C for 1 min before being raised to 320 °C at a rate of 10 °C·min^−1^ and then maintained for 5 min. The injection, transfer line, and ion source temperatures were 280, 280, and 220 °C, respectively. The energy was −70 eV in electron impact mode. The mass spectrometry data were acquired in full-scan mode with the *m/z* range of 85–600 at a rate of 20 spectra per second after a solvent delay of 7.75 min.

### 2.6. Data Analysis

Chroma TOF 4.3X software (LECO Corporation, St. Joseph, MI, USA) and the LECO-Fiehn Rtx5 database were used for raw peak exacting, data baseline filtering, and calibration of the baseline, peak alignment, deconvolution analysis, peak identification, and integration of the peak area [18]. The retention time index (RI) method was used in peak identification and the RI tolerance was 5000. When compounds were identified, the similarity values for the compound identification accuracy are provided. Only when the similarity value was above 700 was the identification of a metabolite considered reliable. Individual integrated peak areas were converted to response ratios relative to the internal standard (l-2-Chlorophenylalanine). Principal component analysis (PCA) and orthogonal projections to latent structures discriminant analysis (OPLS-DA) were performed. Variable influence on projections (VIP) plots in OPLS-DA were used to identify the metabolites that were important for the separation of the study groups. One-way ANOVA was applied to each metabolite. Based on analyses of six biological replicates, the metabolites that had a VIP > 1.0 and *p*-value < 0.05 were considered to be significantly altered metabolites.

## 3. Results

### 3.1. Global Metabolomics Changes in S. furciferainduced by SRBSDV Infection and/or Temperature Stress

**Figure 1:**
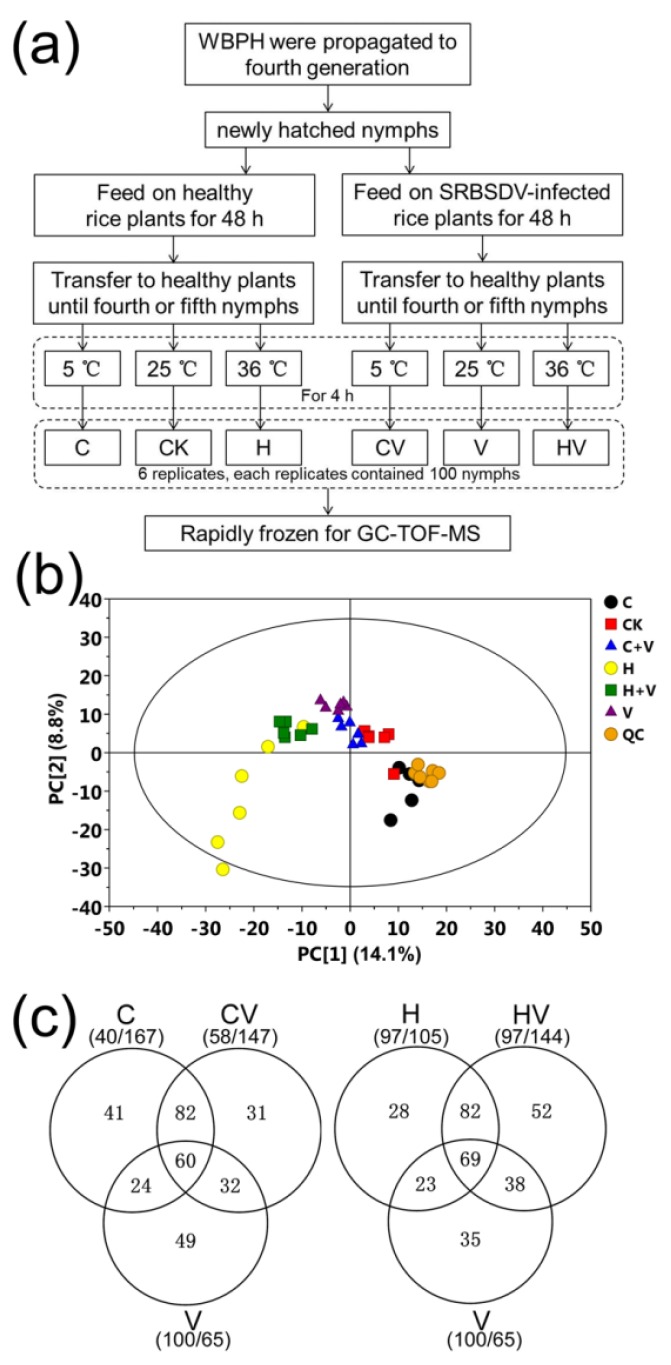
Overview of this metabolomic study. (**a**) Experimental design and work flow of this study. (**b**) Scores plot of the principle components analysis (PCA) model of the gas chromatography-time of flight-mass spectrometry (GC-TOF-MS) data of all samples. (**c**) Venn diagram of the numbers of differentially accumulated metabolites induced by virus infection under cold stress (left) and heat stress (right). Note: CK, the unstressed white-backed planthopper (WBPH) sample; C, cold-stressed WBPH sample; H, heat-stressed WBPH sample; V, *Southern rice black-streaked dwarf virus* (SRBSDV)-carrying WBPH sample; CV, cold-stressed SRBSDV-carrying WBPH sample; HV, heat-stressed SRBSDV-carrying WBPH sample; and QC, quality control sample.

A previous study by our group revealed that SRBSDV infection could decrease the cold tolerance but improve the heat tolerance of its vector WBPH [17]. In this study, comparative metabolomics analysis was used to explore the potential mechanisms underlying these stress responses. Fourth-generation WBPH nymphs were infected with SRBSDV and/or exposed to extreme temperature stress, with virus-carrying or virus-free nymphs grown at 25 °C used as the control (Figure 1a). Metabolomics analyses were used to assess the six groups of treated or control samples. A total of 605 distinguishable peaks were detected and identified, which showed very close retention times (Figure S1). PCA and multivariate data analysis (OPLS-DA) showed that the metabolites of both single and double stress-treated nymphs were clearly differentiated from those of the control samples (Figure 1b). A total of 165, 207, and 202 metabolites differed between the virus infected, cold-, or heat-stressed groups and the control group, respectively (Table S2). For double stress challenged groups, 205 and 241 metabolites differentially accumulated in the virus-carrying groups with cold stress and the virus-carrying group with heat stress, respectively, when compared with the control group (Figure 1c). This indicates that more WBPH metabolites were differentially accumulated under temperature stress than under SRBSDV infection stress.

### 3.2. SRBSDV Infection-Induced S. furcifera Metabolic Changes

By comparing and analyzing the metabolites differentially accumulated between virus-carrying and virus-free WBPH at 25 °C, we found that the majority of differentially accumulated metabolites could be categorized as nucleic acids, sugar and polyols, and fatty acids.

For the nucleic acids metabolites, basic substances (adenine and uracil) and nucleosides (adenosine, guanosine, and inosine) were up-regulated when the WBPH were infected by SRBSDV, whereas the nucleotides (uridine) were down-regulated (Table 1).

SRBSDV infection also led to a change in the energy metabolism of WBPH. The accumulation of the main energy substance trehalose significantly decreased in WBPH infected with SRBSDV, whereas the energy metabolites phosphate and lactic acid both increased.

SRBSDV infection affected the accumulation of fatty acids in WBPH. Myristic acid, oleic acid, and palmitoleic acid were up-regulated, with oleic acid increasing to a very high level. SRBSDV infection led to the accumulation of fatty acids in the insect body; this would provide the material and energy required for the replication and expression of the virus and is, therefore, more conducive to the propagation of the virus in WBPH.

### 3.3. Temperature Stress-Induced S. furcifera Metabolic Changes

Since the tolerance of *S. furcifera* to extreme temperatures is relatively weak, this long-distance migratory insect flies to the tropics for overwintering and then back to high latitudes in the spring [2]. We compared the metabolomic changes of WBPH under temperature stresses.

In cold-stressed WBPH, the most obvious change was that the TCA cycle was altered compared with the control. Pyruvic acid, the substrate of the TCA cycle, increased significantly, whereas levels of the intermediary products citric acid, fumaric acid, and l-Malic acid decreased. Oxalacetic acid and α-ketoglutaric acid levels also increased (Table 2), indicating that virus infection may disturb the aerobic respiration activity of WBPH, thereby affecting the energy supply and slowing the insect growth. These results are consistent with the biological phenomenon wherein WBPH nymphs delayed their development and prolonged developmental duration when grown in cold environments.

Low-temperature treatment also led to the down-regulation of a variety of sugars and polyols in WBPH. Sucrose, 2-deoxyerythritol, 1,5-anhydroglucitol, glucose, and palatinitol levels all decreased at low temperatures, with the first three decreasing markedly compared with the control (Table 2). These small molecules both help to enhance the anti-freeze performance of the biofilms and supply energy for insect activity. These results explained the weak cold tolerance of WBPH at the metabolic level.

In the heat-stressed WBPH, the alteration in the TCA cycle was also the most obvious change which was opposite to the observations with cold treatment; levels of pyruvic acid, the substrate of the TCA cycle, and the intermediary products l-Malic acid, citric acid, and α-ketoglutaric acid were all reduced (Table 3). These trends indicate that high-temperature stress caused an acceleration in WBPH energy metabolism that may help WBPH adapt to hotter environments.

### 3.4. Multiple Stresses Induced WBPH Metabolic Changes

Next, we analyzed the metabolites that responded to SRBSDV infection in combination with temperature stress and found that many amino acids, sugars, and polyols differentially accumulated in comparison with the virus-free WBPH reared at 25 °C.

In WBPH infected with SRBSDV and exposed to cold stress, the levels of 12 amino acids were reduced (Table 4). The downregulation of most of these amino acids was contrary to insects exposed only to cold stress. These results explained the observed biological phenomena where higher death rates of WBPH were observed under cold conditions after SRBSDV infection from a metabolomic perspective. Additionally, levels of seven sugars and polyols, including fructose, sucrose, and trehalose, increased (Table 4). Although these kinds of small molecules may theoretically help improve the cold tolerance of WBPH, the insect body could not cope with the metabolic disorders caused by the strong intensity of viral infection plus cold stress.

In WBPH infected with SRBSDV and exposed to heat stress, most of the amino acids, sugars, and polyols were up-regulated compared with the control (Table 5). The levels of 13 amino acids, including asparagine and threonine, and 10 sugars and polyols all increased with SRBSDV infection and high-temperature treatment. These amino acids induced substances that were important molecules for insect temperature tolerance and may explain the reduced death rates of WBPH under hot condition following SRBSDV infection.

## 4. Discussion

The metabolomic processes of organisms fluctuate in response to many environmental changes. Although these effects may be subtle, they may interfere with the data obtained through GC-TOF-MS. In this study, four generations of WBPH after continuous purification were used as experimental populations to ensure that the genetic background of the individuals tested was relatively consistent. The growth conditions remained stable and insects were transferred to SRBSDV-infected plants for only a short time (48 h) to reduce the indirect effects of rice plants on WBPH metabolism. Additionally, using a large sample group of 100 individuals to obtain metabolomic data would more accurately reflect the effect of virus infection and/or extreme temperatures on the insects. The results showed that the mass spectrum peaks obtained by GC-TOF-MS were well separated and all the distinguishable peaks had very close retention times. Our study identified a wide variety of substances, including amino acids, sugars, polyols, and organic acids, thereby satisfying our aims. In the 36 sets of data obtained from each sample, the retention times of the internal standard in the samples were all about 19 min (Table S1), indicating that the GC-TOF-MS system was very stable. In the PCA of both the whole groups and the two-factor comparison groups, the samples were almost all within the confidence interval of 95% and the samples could be clearly separated. This indicates that the experimental data were reliable, allowing for accurate identification and screening of the differentially accumulated metabolites between the groups.

In addition, these insects are exposed to temperature fluctuations in nature, which means using fluctuations in temperature treatment would better approximate reality. Not only do fluctuations in temperature change the infection efficiency but the temperature can affect the vectorial capacity [19,20]. However, multi-temperature treatment could result in considerably more complicated regulations, which could increase the work required to analyze the data and the increasingly complex relationships. The aim of this study was to identify the potential differential molecules in response to different stress treatments to provide reference and global regulation information about the metabolic pathways in response to different treatments. Therefore, single fixed temperatures were used in this this study to simplify the impact factor, which could not completely mimic or simulate real conditions that these insects encounter in natural conditions. Furthermore, virus infection in insects might also affect the microbiota of these insects, and the microbiota could regulate the virus replication and infection [21]. The virus may also manipulate the microbiota gene expression to indirectly affect the insect vector. Further biological assays are needed to verify the hypothesis.

SRBSDV is transmitted by WBPH in a persistent, circulative, and propagative manner [22], can spread rapidly, and replicate largely within the insect body [23,24]. SRBSDV has a profound impact on insect transcriptomics [17,25] and leads to changes in life parameters, reproductive capacity, and even host choice behaviors [13,14,15,26,27]. In this study, 165 metabolites were identified that were induced by SRBSDV infection at 25 °C when compared with the uninfected control. The results showed that the catabolism of nucleotides was induced following SRBSDV infection, whereas the accumulation of the main energy substance trehalose decreased, and the energy metabolites phosphate and lactic acid increased. This may be caused by the acceleration of the rate of metabolic processes, such as glycolysis, in virus-infected insects that can provide the large amount of energy needed for immune defense. Under extreme temperature treatment, the TCA cycle was the most obvious change that was inhibited under cold treatment and enhanced under heat treatment. These results are consistent with the finding in pea aphids [28], which indicates that the TCA cycle plays an important role in temperature adaption.

Similar to many insect-borne viral diseases, SRBSDV occurs intermittently. This phenotype should not be entirely attributed to human prevention and control, the natural law of which should be further explored. As early as 2009, Sisterson showed that when studying the epidemiology of plant-borne viral diseases, the amount of vector, its transmission rate, and the influence of the disease on the survival rate of vector insects should be considered [29]. SRBSDV has adverse effects on virus-mediated propagation including an increase in nymphal mortality, prolonged nymphal duration, reduced adult life-span, and reduced oviposition [13,14,26]. In our previous study, we hypothesized that the outbreak of SRBSDV inhibited the propagation of WBPH and limited its spread [14]. The increase in WBPH death rates under cold tolerance caused by SRBSDV infection evidently supported this hypothesis. According to the SRBSDV disease cycle, the primary infection is initiated by viruliferous WBPH adults migrating northward from their overwintering sites in warm southern areas every spring [2]. It is generally believed that WBPH need to overwinter at temperatures above 4 °C. A reduction in the cold tolerance of WBPH by SRBSDV infection would inevitably reduce its overwintering area and efficiency and may be an important factor in the inhibition of the spread of the disease.

Inter-biological interactions can produce remarkable biological and ecological effects. Against the background of global climate change, the study of the effects of inter-biological interactions on temperature adaptability has increasingly attracted attention [30,31]. Studies on the effects of parasites on insects have mainly focused on the bacteria- or fungi-induced changes in the extreme temperature tolerance of insects [4,5,6,28,32]. However, few reports have been published about the effects of viruses on the temperature adaptability of their hosts. Many researchers observed that the influence of a virus on the life parameters of insect vectors was temperature dependent [6,14,33]. Therefore, plant viruses may affect the temperature adaptability of their insect vectors if the results were analyzed from a different perspective. Therefore, the effect of SRBSDV on the temperature adaptability of its vector WBPH has extended our understanding of this field.

Previous studies using transcriptome analysis have examined the changes in WBPH under different stresses on the gene expression level [17,25,34]. As with virus infection, extreme temperatures, or insecticide treatment, each stress could lead to the change in the expression patterns in WBPH, and these results provide a valuable resource for the molecular characterization of stress action in WBPH [17,25,34]. Moreover, Than et al. investigated the protein–protein interactions between SRBSDV and WBPH [35]. They identified over 100 WBPH proteins as putative interactors of the coat protein of SRBSD, which helped increase the understanding about how SRBSDV affects its insect vector. In this study, based on our previous transcriptome analysis, a metabolomics study was used to investigate the mechanism underlying this phenomenon. The results indicated that SRBSDV may influence the extreme temperature tolerance of WBPH by regulating the accumulation of amino acids, sugars, and polyols in the insect body. The amino acids, including methionine, proline, threonine, ornithine, l-homoserine, and β-alanine, were down-regulated with SRBSDV infection combined with cold stress and were up-regulated in SRBSDV infection combined with heat stress. The sugars and polyols were up-regulated in both these groups. These molecules may play an important role in influencing the temperature adaptability of WBPH. Additional investigations are needed to further characterize this mechanism and to provide new strategies for the prevention and control of SRBSDV based on this mechanism.

## 5. Conclusions

In summary, this study was completed from the perspective of an insect virus. We analyzed the metabolomic changes in WBPH in response to SRBSDV infection (biotic stress) and/or extreme temperature (abiotic stress) to characterize the mechanism underlying virus-mediated changes in the temperature adaptability of the insect vector. Our conclusions provide theoretical knowledge for the prevention and control of SRBSDV transmitted by WBPH.

## Figures and Tables

**Table 1:** Major metabolite changes in white-backed planthopper (WBPH) with *Southern rice black-streaked dwarf virus* (SRBSDV infection). By gas chromatography-time of flight-mass spectrometry (GC-TOF-MS) analyses of six biological replicates of virus-carrying and virus-free WBPH at 25 °C, the metabolites that had a variable influence on projections (VIP) > 1.0 and *p*-value < 0.05 were considered to be significantly altered metabolites. Nucleic acids, sugars and polyols, and fatty acids were selected to show in the table.

Category	Peak	Similarity	VIP	*p*-Value	Fold Change
Nucleic acids	Adenine	753	1.35	0.019	1.17
	Adenosine	812	1.84	2.91 × 10^–5^	1.17
	Uracil	968	1.48	0.007	1.05
	Guanosine	813	1.61	0.002	1.10
	Inosine	909	1.73	2.82 × 10^–4^	1.09
	Uridine	688	1.80	5.14 × 10^–5^	0.76
Sugars and polyols	Trehalose	946	2.00	6.64 × 10^–13^	0.65
	Phosphate	772	1.39	0.025	2.31 × 10^7^
	Lactic acid	967	1.59	0.003	1.14
Fatty acids	Myristic acid	846	1.34	0.023	1.11
	Oleic acid	863	1.68	0.004	3.27 × 10^6^
	Palmitoleic acid	902	1.18	0.044	1.05

**Table 2:** Major metabolite changes in WBPH under cold stress. By GC-TOF-MS analyses of six biological replicates of cold-stressed and unstressed virus-free WBPH, the metabolites that had a VIP > 1.0 and *p*-value < 0.05 were considered to be significantly altered metabolites, and the metabolites involved in TCA cycle, sugars, and polyols were selected to show in the table.

Category	Peak	Similarity	VIP	*p*-Value	Fold Change
TCA cycle	Oxalacetic acid	470	1.67	7.21 × 10^–4^	1.90
	Pyruvic acid	926	1.59	1.50 × 10^–4^	1.50
	α-Ketoglutaric acid	910	1.75	1.36 × 10^–8^	1.44
	Citric acid	881	1.24	0.015	0.95
	Fumaric acid	654	1.19	0.017	0.78
	l-Malic acid	888	1.78	2.92 × 10^–10^	0.55
Sugars and polyols	Glucose	336	1.08	0.032	0.82
	Palatinitol	411	1.34	6.60 × 10^–3^	0.82
	Sucrose	778	1.79	6.52 × 10^–7^	3.22 × 10^–5^
	2-Deoxyerythritol	785	1.76	3.25 × 10^–8^	0.47
	1,5-Anhydroglucitol	436	1.55	1.69 × 10^–5^	0.07

**Table 3:** Major metabolite changes in WBPH under heat stress. By GC-TOF-MS analyses of six biological replicates of heat-stressed and unstressed virus-free WBPH, the metabolites that had a VIP > 1.0 and *p*-value < 0.05 were considered to be significantly altered metabolites, and the metabolites involved in TCA cycle were selected to show in the table.

Category	Peak	Similarity	VIP	*p*-Value	Fold Change
TCA cycle	Pyruvic acid	926	1.34	0.006	0.77
	α-Ketoglutaric acid	910	1.66	1.44 × 10^–5^	0.77
	Citric acid	881	1.76	3.14 × 10^–10^	0.29
	l-Malic acid	888	1.76	3.14 × 10^–10^	0.25

**Table 4:** Major metabolite changes in SRBSDV-infected WBPH under cold stress. By GC-TOF-MS analyses of six biological replicates of cold-stressed and unstressed virus-carrying WBPH, the metabolites that had a VIP > 1.0 and *p*-value < 0.05 were considered to be significantly altered metabolites, and amino acids, sugars, and polyols were selected to show in the table.

Category	Peak	Similarity	VIP	*p*-Value	Fold Change
Amino acids	Asparagine	911	1.73	6.98 × 10^–7^	0.70
	Glutamine	891	1.24	0.020	0.64
	Isoleucine	962	1.64	3.64 × 10^–5^	0.87
	Lysine	865	1.79	7.64 × 10^–10^	0.83
	Methionine	865	1.64	4.88 × 10^–5^	0.87
	Phenylalanine	943	1.74	1.22 × 10^–7^	0.84
	Proline	596	1.41	0.003	0.96
	Threonine	957	1.69	3.85 × 10^–6^	0.87
	Tryptophan	897	1.78	9.77 × 10^–11^	0.81
	Ornithine	918	1.72	1.06 × 10^–6^	0.82
	l-homoserine	918	1.41	0.002	0.94
	β-Alanine	925	1.79	4.85 × 10^–12^	0.58
Sugars and polyols	d-talose	929	1.73	1.70 × 10^–6^	1.23
	Fructose	958	1.72	1.82 × 10^–6^	1.12
	Fructose-6-phosphate	753	1.68	1.24 × 10^–5^	1.35
	Sucrose	778	1.61	4.21 × 10^–5^	1.30
	Tagatose	725	1.25	0.010	1.28
	Trehalose	946	1.77	1.81 × 10^–8^	1.15
	Glycerol	823	1.70	2.56 × 10^–6^	1.17

**Table 5:** Major metabolite changes in SRBSDV-infected WBPH under heat stress. By GC-TOF-MS analyses of six biological replicates of heat-stressed and unstressed virus-carrying WBPH, the metabolites that had a VIP > 1.0 and *p*-value < 0.05 were considered to be significantly altered metabolites, and amino acids, sugars, and polyols were selected to show in the table.

Category	Peak	Similarity	VIP	*p*-Value	Fold Change
Amino acids	Alanine	800	1.67	2.11 × 10^–11^	1.32
	Asparagine	911	1.65	3.86 × 10^–9^	1.58
	Glutamic acid	866	1.62	6.20 × 10^–7^	1.37
	Glycine	860	1.46	1.78 × 10^–4^	1.23
	Methionine	865	1.61	1.04 × 10^–6^	1.21
	Proline	596	1.66	4.35 × 10^–10^	1.21
	Serine	964	1.15	0.015	1.05
	Threonine	957	1.67	9.44 × 10^–11^	1.74
	Valine	880	1.62	6.08 × 10^–7^	1.27
	β-Alanine	925	1.66	7.49 × 10^–9^	1.42
	Norvaline	614	1.57	5.50 × 10^–6^	1.45
	Ornithine	945	1.59	2.89 × 10^–6^	1.39
	l-homoserine	918	1.65	1.99 × 10^–8^	1.39
Sugars and polyols	Allose	589	1.43	6.02 × 10^–4^	3.12
	d-glucoheptose	601	1.47	1.67 × 10^–4^	4.19
	d-talose	929	1.61	6.77 × 10^–7^	1.25
	Fructose	958	1.47	2.24 × 10^–4^	1.08
	Lactose	547	1.05	0.042	1.79
	Trehalose	946	1.24	0.029	1.22
	Lactulose	488	1.09	0.022	1.87
	2-Deoxyerythritol	785	1.27	0.005	1.15
	1,5-Anhydroglucitol	436	1.09	0.026	1.12
	Glycerol	823	1.05	0.030	1.04

## Supplementary Materials

The following are available online at http://www.mdpi.com/1999-4915/10/7/344/s1, Figure S1: WBPH viruliferous rate and internal standard retention time of the 36 samples. Table S1: WBPH viruliferous rate and internal standard retention time of the 36 samples. Table S2: The accumulated amounts of each metabolite in each treatment.
